# Quantification of lactobionate using ion-exclusion high-performance liquid chromatography

**DOI:** 10.3168/jdsc.2026-1136

**Published:** 2026-09-07

**Authors:** Yufeng Lin, Usman Amin, C.K. Yeung, Haotian Zheng

**Affiliations:** 1 Food Rheology Laboratory, Department of Food, Bioprocessing and Nutrition Sciences, North Carolina State University, Raleigh, NC 27695; 2 Southeast Dairy Foods Research Center, North Carolina State University, Raleigh, NC 27695; 3 Animal Science Department, California Polytechnic State University, San Luis Obispo, CA 93407

## Abstract

Lactobionic acid (LBA) and calcium lactobionate (Ca-LBN) are emerging value-added ingredients that can be manufactured from cheese whey permeate streams. Reliable analytical methods for quantifying the contents of LBA or lactobionate (LBN) in processed products derived from dairy permeates are still scarce. This study established a rapid ion-exclusion high-performance liquid chromatography (IE-HPLC) method for quantifying LBN and lactose in a product slurry obtained from enzymatic lactose oxidation trials for manufacturing Ca-LBN from cheese whey permeate. Chromatographic separation was achieved using an ion exclusion column containing fully sulfonated resin with an aqueous H_2_SO_4_ mobile phase (0.45 m*M*) at 0.5 mL/min and 75°C. The chromatogram analysis was assisted by the Gaussian deconvolution method for resolving overlapping peaks between the LBA anion and lactose. Standard curves of LBA and lactose standards were prepared within the same concentration range of 0.5 to 10 m*M*. The IE-HPLC method was able to quantify LBN anion presence in the Ca-LBN slurry as processed from cheese whey permeate through the mentioned enzymatic oxidation process. According to the IE-HPLC analysis, the LBN anion content in the Ca-LBN slurry was characterized as ∼755 m*M*; this was equivalent to an 86% lactose-LBN molar conversion rate (given the original lactose substrate content was 300 g/L ≈ 877 m*M*). This was an underestimation of ∼13% compared with the previously reported conversion rate (∼99%) in another publication for the same sample, in which the conversion rate was calculated based on Ca(OH)_2_ consumption during pH-stat auto-titration in response to produced LBN anions. Such a deviation in LBN quantification between the IE-HPLC method used in the present work and the pH-stat method used in the other work suggested that the IE-HPLC analysis might result in underestimation of the absolute quantity of LBN or LBA anions. This was confirmed by the spike recovery experiment. The Ca-LBN sample was spiked with an additional LBA standard, and the spiked Ca-LBN did not show an additional peak in the chromatogram after Gaussian deconvolution peak fitting, validating the LBN anion peak identification. Moreover, the spike recovery was ∼89%, confirming that IE-HPLC may underestimate LBN or LBA in processed dairy permeate. This documented IE-HPLC method can be used by industry, including LBN manufacturers and LBN ingredient users, for quality control and for quantity validation purposes.

## Graphical Abstract

**Figure:**
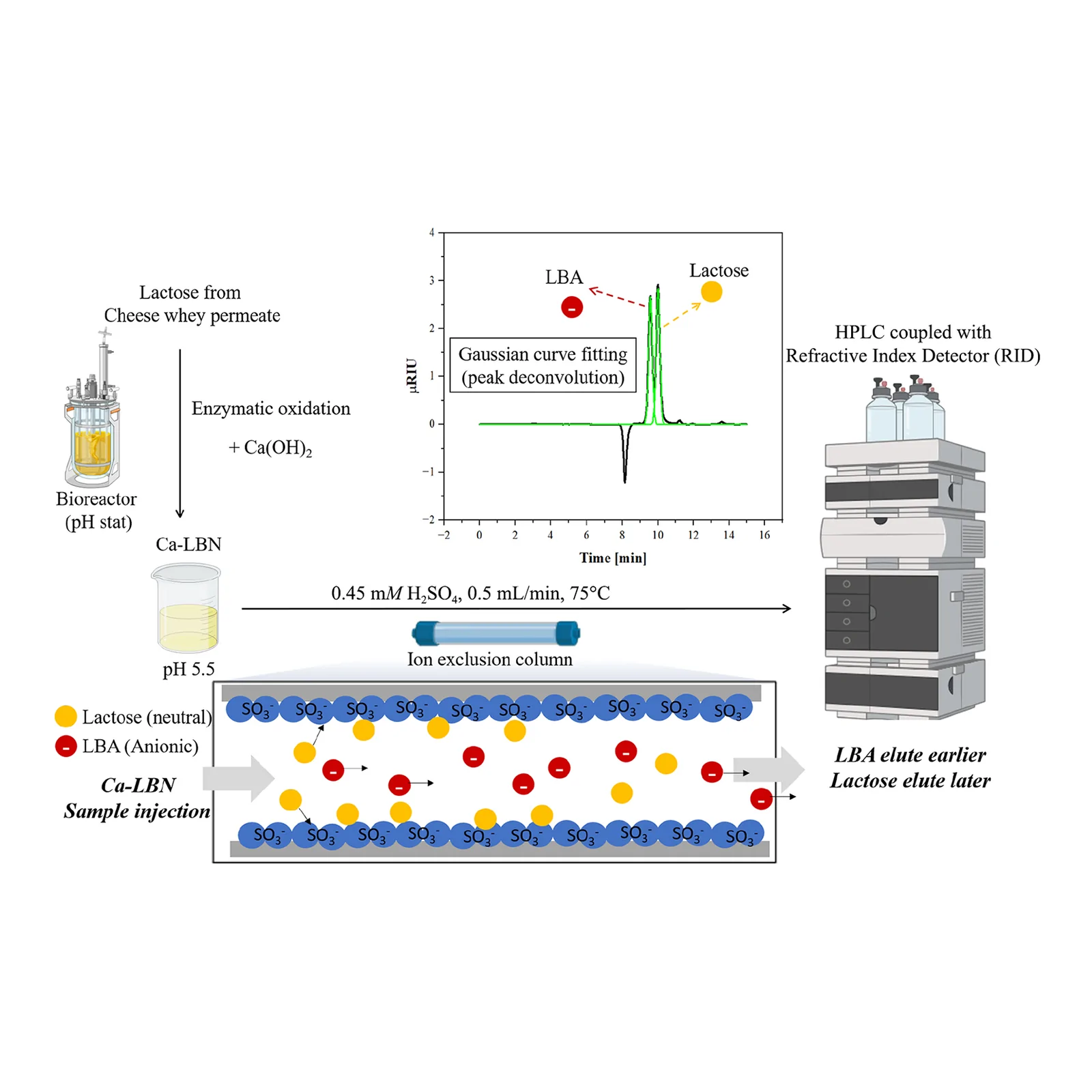

Lactobionic acid (**LBA**; 4-Ο-β-D-galactopyranosyl-D-gluconic acid) and its salt form, lactobionate (**LBN**), may be used in food, medical, and cosmetics applications (Isaacson et al., 1989; Cardoso et al., 2019; Tasić-Kostov et al., 2019). Lactobionic acid is a polyhydroxy acid derived from lactose oxidation and exhibits excellent hygroscopicity, metal chelation, and antioxidant properties (Alonso et al., 2013; Cardoso et al., 2019). These properties make LBA and LBN value-added ingredients as compared with lactose. This motivated research works focused on developing and improving different approaches for manufacturing LBA and LBN derived from the permeate streams from the dairy foods industry (e.g., cheese whey permeates; Hua et al., 2007; Tasic-Kostov et al., 2012; Amin et al., 2025). Such activities may support valorization of whey permeate and ultimately improve the utilization and net value of milk solids. Calcium lactobionate (**Ca-LBN**) is of more interest compared with other forms of LBN because the calcium salt provides additional nutritional and functional advantages for food and pharmaceutical applications (Cardoso et al., 2019). More importantly, Ca-LBN is approved as a food ingredient by the US Food and Drug Administration (FDA, 2018).

The enzymatic oxidation process as a green technology has been conducted for producing LBN, the anion part of LBA, from lactose; the enzymatic approach has a few advantages, such as mild processing conditions and high molar conversion rate (Nordkvist et al., 2007; Amin et al., 2025). This process is typically based on the pH-stat method; during the process, a commercial cellobiose oxidase (e.g., LactoYIELD, Chr. Hansen), typically coupled with catalase, converts the lactose into LBA. According to Amin et al. (2025), a base solution (e.g., Ca(OH)_2_) is added to the reaction system to maintain pH at a level (pH 6.4) in the range of 5.5 to 7.0 according to the suggestion from the oxidase manufacturer; the dissociation constant (**p*K*_a_**) of LBA is ∼pH 3.8; therefore, at the processing pH 6.4, the LBN anion form dominated in the product system. Such an enzymatic conversion processing method is effective. For instance, Amin et al. (2025) showed that a pH-stat–based enzymatic oxidation process resulted in ∼99% molar conversion of lactose from cheese whey permeates (∼300 g/L) to Ca-LBN within 7 h. Although the method was scalable and feasible with a high-concentration substrate system, the quantification of LBN being processed was based on an indirect titration-calculation method according to the volume of a titrant solution (e.g., Ca(OH)_2_) consumed during the processing course. Such a quantification method is only suitable for monitoring LBN production during processing, and it cannot be used for validating or quantifying LBN content in a commercial ingredient in either liquid or powder form. It is important to note that a Ca-LBN dispersion derived from an enzymatic oxidation as described in Amin et al. (2025) will need to be further processed through evaporation and crystallization to be able to obtain the powder form of LBN ingredient for different industrial applications. Therefore, for the sake of quality assurance and quality control of LBN ingredients regardless of their physical form (liquid or dried powder), a direct analytical method for LBN anion quantification, alternative to the mentioned titration-calculation method, is essential for industrial operation. High-performance liquid chromatography coupled with a refractive index detector (**RID**) has been widely used for carbohydrate and organic acid analyses. The separation and quantification of lactose and LBN using HPLC is challenging due to their structural similarity and lack of strong UV chromophores, requiring refractive index or electrochemical detection. Earlier studies have explored HPLC methods for such separation. Simms et al. (1994) achieved complete resolution of lactose, lactobionic acid, and the related lactone using specialized columns (β-cyclodextrin-bonded and aminopropyl-silica phases); however, their work did not provide quantification information. Moreover, according to Simms et al. (1994), a cation-exchange column was able to separate between LBA and lactose, whereas lactose and lactobionolactone showed a co-elute effect. The objective of the present work is to document an analytical HPLC method based on cation-exclusion separation for quantifying the LBN content in sample slurries, which were obtained as products from enzymatic oxidation processing trials. Cheese whey permeate was used as the substrate in the referred LBN processing experiments. This work also attempted to address the quantification challenge caused by partial peak overlap through the application of a Gaussian deconvolution approach. The LBA (97%) standard sample was sourced from Thermo Scientific Chemicals (Waltham, MA). Sulfuric acid (Certified ACS Plus, 99.9%) and calcium hydroxide (Ca(OH)_2_, 95%) were acquired from Thermo Scientific Chemicals (Waltham, MA). The cheese whey permeate ingredient (DAIRYLAC 80-16, International Ingredients Inc.) contained 82.52% lactose, 2.82% protein, 1.42% moisture, and 0.25% water activity and was kindly provided by Hilmar Cheese Company (Hilmar, CA). Water used for HPLC analyses was purified to 18.2 MΩ·cm Type I ultrapure water using a PURELAB Classic system (ELGA LabWater, Woodridge, IL). All samples were filtered using 0.45-μm pore size syringe filters (diameter 33 mm, hydrophilic polyethersulfone membrane) sourced from MilliporeSigma (St. Louis, MO).

The Ca-LBN dispersion for HPLC analysis used in this study was the sample product manufactured from our previous work regarding the establishment of an in-house enzymatic oxidation process for LBN production (Amin et al., 2025). The detailed bioreactor configurations, processing parameters, and substrate-product molar conversion rate can be found in Amin et al. (2025). Before HPLC injection, the sample was diluted using deionized (DI) water to bring lactose and lactobionate concentrations within the analytical range and to reduce viscosity. The sample was also filtered through a 0.45-µm membrane to remove any precipitates or cell debris (the enzymatic process produces no biomass, but CaCO_3_ or other colloids could form).

Chromatographic separation was performed on a Vanquish Core HPLC system equipped with a quaternary pump, autosampler, and temperature-controlled column compartments (Thermo Fisher Scientific, Germering, Germany); an RID (RefractoMax521, IDEX Health & Science, Saitama, Japan) was used for the detection of target analytes eluted from the stationary phase. An ion-exclusion (**IE**) column (Waters IC-Pak Ion-Exclusion Column, 7 µm, 7.8 mm × 300 mm, maximum pressure 140 bar) was used as the stationary phase; it was kept at 75°C during the HPLC separation process. The column contained fully sulfonated (cation exchange) resin. A 0.45 m*M* H_2_SO_4_ solution (at pH 3.1) was used as the mobile phase with an elution flow rate of 0.5 mL/min. Preliminary experiments were conducted to select the mentioned column temperature and the mobile phase pH according to the characteristics of chromatogram peaks of the analytes. The RI detector was operated at 35°C. Each measurement was conducted using an isocratic condition with a typical running time of approximately 20 to 25 min per injection. The column was cleaned by flushing it with the sulfuric acid mobile phase. Adequate removal of residual solutes was confirmed by injecting a water blank after each sample and verifying the absence of carryover peaks.

The standard solutions for lactose and LBA were prepared to obtain a concentration range of 0.5 to 10 m*M*. Five specific concentrations within the mentioned range (0.5, 1, 2.5, 5, 10 m*M*) were used. The LBA standard solution was prepared according to previous work with modifications (Pedruzzi et al., 2007). Regarding the preparation of LBA standard solutions, briefly, the LBA was dissolved in a 20 m*M* Ca(OH)_2_ solution to obtain 20 m*M* LBA content and a pH within a range of 11 to 12. The resulting solution was topped up with sulfuric acid mobile phase (at pH 3.1) and DI water to reach a target concentration as a stock solution (10 m*M*). The final pH was controlled at ∼5.5 as the working pH. The stock solution was diluted to the mentioned standard concentrations using DI water.

Figure 1 shows the chromatograms of individual standards of LBA (Figure 1A) and lactose (Figure 1B). Injection of the LBA standard produced a distinct peak with a retention time of ∼9.7 min, whereas the lactose standard eluted slightly later at ∼10.0 min. An acceptable linearity was observed for the 2 standard curves, respectively, with coefficients of determination (R^2^) greater than 0.99 (Figure 1D). These results confirmed that the detector responses were linear within the standard concentration ranges used in this study.

**Figure 1:**
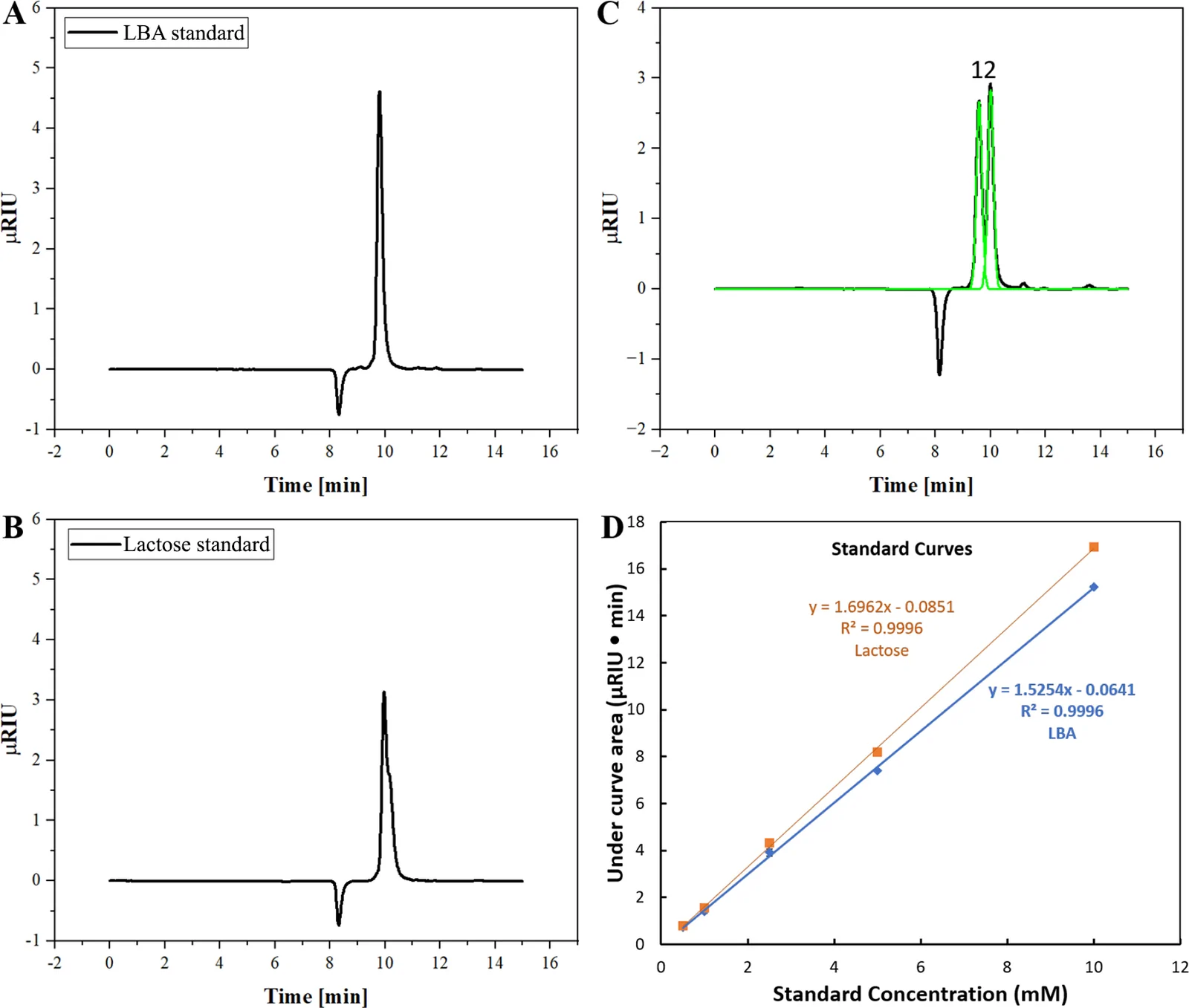
(A) Chromatogram of lactobionic acid (LBA) standard solution (1 m*M*). (B) Chromatogram of lactose standard solution (0.5 m*M*). (C) Chromatogram of a mixture solution containing LBA and lactose (0.5 m*M* for each analyte; 1: LBA, 2: lactose). The dark-colored curve was the original chromatogram containing overlapped peaks; the green-colored curve was the processed chromatogram after Gaussian fitting. The latter illustrates peak separation for quantification purposes. (D) Standard curves including linear trendline equations and their coefficients of determination (R^2^) of LBA (blue color) and lactose (orange color). The standard deviations of the characterized under curve areas for both LBA and lactose standards are within a range of 0.01–0.16 (μRIU/min). RIU = refractive index unit.

However, for a mixed standard solution containing both analytes, overlapped peaks were observed in the chromatogram, as shown in Figure 1C. A similar peak overlapping phenomenon between lactose and LBA was reported for cation-exchange chromatography in a previous work (Pedruzzi et al., 2007). An IE column containing fully sulfonated resin was used in the present study; part of the separation mechanism was based on Donnan exclusion, in which differential partitions of solutes between the mobile eluent and the immobilized eluent in the (charged) sulfonated resin were used for separation of differently charged molecules (Fritz, 1991). The p*K*_a_ value of LBA is ∼pH 3.8 according to the literature (Green et al., 2009; Bhattacharyya and Rohrer, 2012). Therefore, a higher value of an acidic eluent pH (e.g., 5 > pH > 3.8) would be favored for lactose and LBA separation using the referred IE column because in this pH range, LBA was predominantly in deprotonated form (anion of LBA) carrying negative charge and lactose carried zero net charge. Therefore, LBA would elute faster than lactose. However, it was shown in a previous work that an eluent at >pH 3.1 (e.g., ∼4) had resulted in an asymmetry-shaped LBA peak, whereas the LBA peak shape was shown as symmetric at pH 3.1 while kept separate from the lactose peak (Pedruzzi et al., 2007). However, the complete separation between the LBA and lactose peaks was not seen in the chromatogram shown in Figure 1C; such a slight deviation compared with the reference might be attributed to the different columns used between the 2 studies. Although the columns used between the 2 studies were all IE columns and had comparable configurations, they were produced by different manufacturers. Theoretically, at pH 3.1, both LBA and lactose are predominantly in protonated forms, whereas lactose still has a higher cation exclusion effect than LBA due to its dramatically higher p*K*_a_. Therefore, lactose eluted slightly later than LBA, as shown in Figure 1. In summary, adjusting environmental pH might not be an ideal approach for improving the peak separation due to a higher pH value in the acidic range that would deform the LBA peak shape.

The peak overlap effect may negatively affect the accuracy of LBA and LBN (anion) quantification using the established LBA standard curve mentioned previously. The conventional peak integration by perpendicular drop at the valley can introduce systematic errors by misallocating the signal in the overlap region. Therefore, a mathematical approach instead of chemistry approach was attempted for resolving this analytical challenge. Specifically, a peak deconvolution method was conducted for analyzing the peaks that resulted from the 2 analytes. Gaussian curve fitting was employed to deconvolute the overlapping peaks. Briefly, the chromatograms were analyzed using OriginPro software (OriginPro 2023b, OriginLab Corp., Northampton, MA), where Gaussian fitting was employed to determine the peak deconvolution: the overlapping region of the chromatogram was fitted to the sum of 2 Gaussian curves, corresponding to the LBA and the lactose peaks. Initial parameters (peak center and width) for the fitting were obtained from the separate injections of each standard. The fitting was iteratively optimized to minimize residuals, yielding individual peak areas for each component. The area under each Gaussian curve was taken as the quantitative measure of that component. This approach assumes symmetric peak shapes; in practice, slight tailing was observed, but the Gaussian model was found to be adequate, as discussed later.

The Ca-LBN sample slurry produced by the enzymatic oxidation of lactose was expected to contain lactobionate as the dominant species with potentially minor residual lactose according to the reported molar conversion results derived from pH-stat–based calculation, as shown in our previous work (Amin et al., 2025). The Ca-LBN sample dilutions (with a 200× dilution) were analyzed using HPLC in triplicate. As shown in Figure 2A, the chromatogram of the diluted Ca-LBN sample showed a single dominant peak corresponding to the retention time of the anion part of lactobionate, suggesting a near-complete molar conversion of lactose. This result is consistent with the high conversion efficiency reported by the reference, in which we reported ∼99% conversion of lactose to LBN (Amin et al., 2025). Importantly, the mentioned conversion rate was only an estimate, which was indirectly calculated according to the base solution consumption. The present study provided more direct evidence confirming the high conversion rate using HPLC analysis. According to the fitted peak area shown in Figure 2A and the developed standard curve of LBA (Figure 1D), the molar content of LBN anions in the original Ca-LBN was characterized as ∼755 m*M*; this is equivalent to a lactose-LBN molar conversion rate of ∼86% given that the Ca-LBN sample originated from a substrate containing 300 g/L lactose (equivalent to ∼877 m*M*) according to Amin et al. (2025). Amin et al. (2025) reported that the lactose-LBN molar conversion rate was ∼99%; such an estimate was obtained by calculation using data from a pH-stat auto-titration method. Therefore, the Ca-LBN conversion rate as characterized by the HPLC method was underestimated by ∼13% compared with the rate obtained from the pH-stat method. Such a deviation might be attributed to the underestimated peak area due to Gaussian model fitting, which is discussed later regarding spike recovery analysis. Therefore, the presented HPLC method can serve as a quality control and validation method for testing the lactose-LBN conversion efficiency for future industrial production of Ca-LBN using the enzymatic oxidation process.

**Figure 2:**
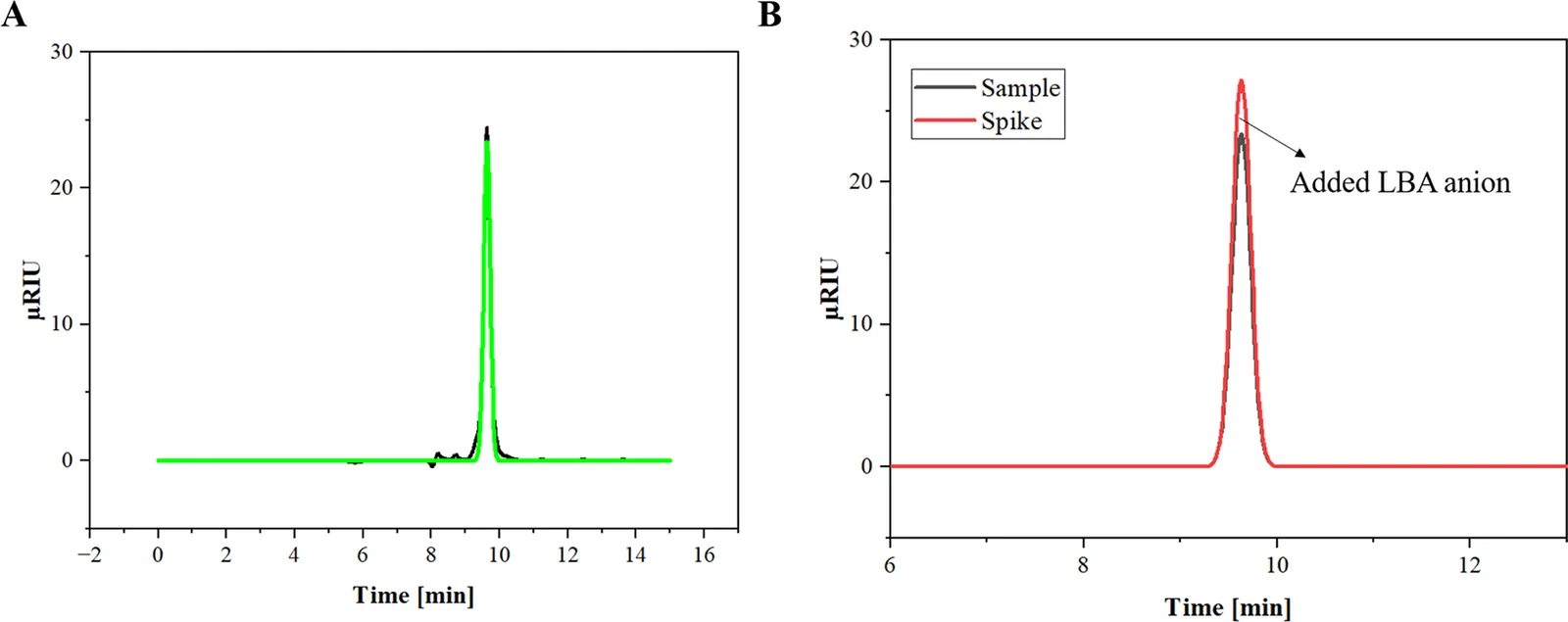
(A) Chromatogram of the manufactured Ca-LBN sample solution (diluted to a concentration to fit within the range of LBA and lactose standard curves). The dark chromatogram was derived from the HPLC measurement of the diluted Ca-LBN sample; the green curve was derived from Gaussian fitting of the original chromatogram. (B) Gaussian-fitted chromatograms of the diluted Ca-LBN sample and the Ca-LBN sample spiked with added LBA standard. All peaks were subtracted from the baseline, and the peak fitting was performed based on a Gaussian fit for calculating the under-peak areas. The baseline was prepared from the mobile phase solution alone. RIU = refractive index unit.

It is interesting to note that the retention time of the anion of LBN in the diluted Ca-LBN sample was shifted to a slightly earlier time (Figure 2A) compared with the LBA standard (Figure 1A). To assess the accuracy of the HPLC-based LBN quantification method and to confirm the identified major peak shown in Figure 2A as the anion of LBN, a spike recovery test was performed. A known quantity of the LBA standard was added to a portion of the Ca-LBN solution (before dilution), and the sample was then diluted and analyzed. The LBN anion percent recovery was calculated as follows:

$$Spikerecovery\left(\%\right)=\frac{LBN_{spike}-LBN_{original}}{LBA_{spike}}\times 100\%,$$

where *LBN_spike_* and *LBN_original_* are the measured LBN anion amounts in the LBN sample spiked by additional LBA and the original LBN sample, respectively, and *LBA_spike_* is the amount of added LBA.

In addition, the reproducibility of the analysis was evaluated by 3 repeated injections of the same sample; the relative standard deviation of the measured lactobionate and lactose concentrations was <2%. No significant carryover in the column between measurements was observed, as the blank water injections after selected sample runs showed negligible peaks.

Regarding the spike recovery analysis, briefly, a known amount (0.0358 g) of LBA standard was then added to the original Ca-LBN slurry derived from the enzymatic oxidation production trial to yield a spiked Ca-LBN sample. Both the original and the spiked samples were diluted and analyzed using the HPLC method described previously. Chromatographic peaks were processed using baseline subtraction followed by Gaussian peak fitting to resolve the partially overlapping lactose and lactobionate signals, as shown in Figure 2B. According to Figure 2B, the spiked Ca-LBN sample did not show an additional segregated peak; instead, it only showed an increased peak area compared with the Ca-LBN control. This suggested that the shown major peak was indeed the anion of LBN or LBA, and the added LBA resulted in a positive response in the HPLC measurement. The concentration of LBN in the spiked sample was calculated using the aforementioned standard curve of LBA, and the spike recovery (%) was determined using the equation described previously. The spike recovery was found at ∼89.33%.

The reported spike recovery (<100%) may be attributed to several factors. For instance, peak tailing and peak asymmetry can lead to partial loss of integrated areas if the asymmetric areas were not fully captured during peak fitting. This effect may persist even when Gaussian deconvolution is applied, particularly if the tail extends beyond the truncation limits of the fitting window (Wahab et al., 2019). Figures 1A and 1B show that both LBA and lactose standard peaks presented moderately asymmetric shapes. These not only explained the lowered spike recovery, but also the underestimated molar content of Ca-LBN based on this HPLC analysis coupled with Gaussian fitting as compared with the pH-stat estimation method. Moreover, the Ca^2+^ derived from the Ca-LBN sample might have interacted with the negatively charged resins in the cation-exclusion column; in addition, adding external standard (LBA) in the LBN sample might result in a slight modification of the refractive index. This may cause matrix interferences and affect the elution of LBN anions. Future research efforts are recommended for further improving the spike recovery, the quantification accuracy, and the chromatographic separation between LBN and lactose. Some possible approaches worth trying in the future are (1) reducing injection volume or analyte concentration to minimize column overloading and improving peak symmetry (Dong, 2006) and (2) adjusting the ionic strength or composition of the mobile phase for modulating analyte selectivity or analyte partition behavior between the stationary and mobile phases (Lee, 1990).

In summary, an IE–based HPLC method coupled with Gaussian deconvolution for resolving partially co-eluted peaks was established in-house for the quantification of Ca-LBN in a product slurry derived from cheese whey permeate as the substrate; the product was obtained from our previous work involving laboratory-scale bioreactor processing trials of enzymatic lactose oxidation. Using an IE column containing fully sulfonated resin coupled with refractive index detection and Gaussian peak deconvolution, the integrated method enabled effective quantification of LBN product in a real sample slurry originating from enzymatic oxidation trials. This suggested that the reported analytical method can be implemented for testing and validating LBN quantity in a product slurry derived from the same enzymatic processing approach, although the HPLC method resulted in a slight underestimation. Direct chromatographic quantification confirmed near-complete lactose-to-LBN molar conversion, providing an analytical validation of the high-yield enzymatic production protocol reported by Amin et al. (2025). The identification of the LBN anion peak in the Ca-LBN sample was validated by an LBA spike added to the Ca-LBN sample; moreover, the spike recovery was characterized at ∼89%. Future research is recommended to characterize the extent of calcium and cation-exchange resin interactions within an IE column and investigate the impact of calcium-resin interaction on resolution of lactose and LBN and LBN quantification. Overall, this work established a feasible analytical method for quantifying anions of LBN and LBA in a processed LBN product slurry; it can serve for quality control purposes of LBN products derived from future industrial manufacturing processes.
